# 910. Impact of Indication-based Antibiotic Order Sentences on Optimal Prescribing in the Emergency Department: A Quasi-experiment

**DOI:** 10.1093/ofid/ofac492.755

**Published:** 2022-12-15

**Authors:** Lisa Vuong, Rachel M Kenney, Julie Thomson, Darius J Faison, Michael P Veve

**Affiliations:** Henry Ford Hospital, Detroit, Michigan; Henry Ford Hospital, Detroit, Michigan; Henry Ford Hospital, Detroit, Michigan; Henry Ford Wyandotte Hospital, Detroit, Michigan; Eugene Applebaum College of Pharmacy and Health Sciences, Detroit, Michigan

## Abstract

**Background:**

A prior study demonstrated suboptimal antibiotic prescribing in the emergency department (ED) at 77.4% for uncomplicated lower respiratory tract infections (LRTI), urinary tract infections (UTI), and acute bacterial skin and skin structure infections (ABSSSI). This study measured the effect of indication-based antibiotic order sentences (AOS) on prescribing.

**Methods:**

IRB-approved quasi-experiment of adults prescribed antibiotics in ED for uncomplicated LRTI, UTI, or ABSSSI from January - June 2019 (pre-group) or September - December 2021 (post-group). Exclusion: hospital admission, immunocompromised, active cancer, or prophylactic antibiotics. AOS are lean process, electronic discharge prescriptions retrievable by name or indication; AOS implementation occurred July 2021. Optimal prescribing was defined as the correct antibiotic selection, dose, and duration per local and national guidelines. Seven-day endpoints: antibiotic escalation, ED or hospital readmission, any outpatient contact, and reported adverse drug event (ADE). Descriptive and bivariate statistics performed. Variables considered for multivariable logistic regression had p< 0.2 or plausible association with optimal prescribing.

**Results:**

294 patients included: 147-pre and 147-post. Patient characteristics are in Table 1. Overall optimal prescribing improved from 12 (8.2%) to 34 (23.1%) (p< 0.001). Breakdown of optimal prescribing in pre- and post-groups: selection 90 (61.2%) vs 117 (79.6%) (p< 0.001), dose 99 (67.3%) vs 115 (78.2%) (p=0.036), duration 38 (25.9%) vs 50 (34%) (p=0.126). After adjustment, AOS were independently associated with optimal prescribing (Table 2). Secondary endpoints: antibiotic escalation 10 (6.8%) vs 7 (4.8%) (p=0.662), hospital or ED readmission 12 (8.2%) vs 10 (6.8%) (p=0.658), outpatient contact 31 (21.1%) vs 28 (19%) (p=0.662), and ADE 2 (1.4%) vs 4 (2.7%) (p=0.684). Post-hoc analysis showed suboptimal uptake of AOS by ED prescribers.

**Table 1:**
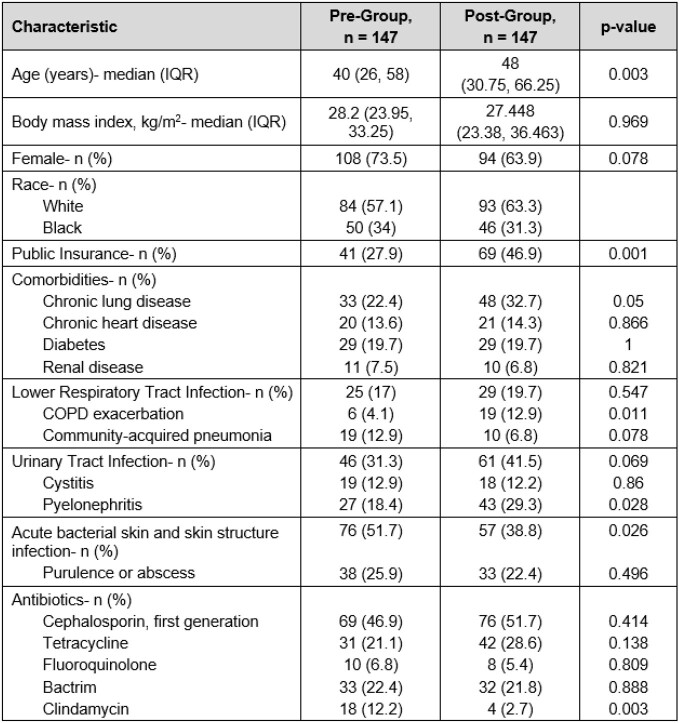
Baseline and Clinical Characteristics

**Table 2:**
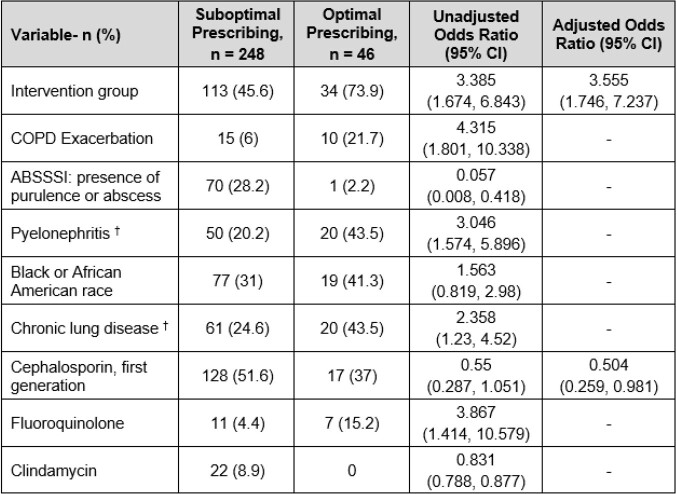
Bivariable and Multivariable Regression Analysis of Factors Associated with Optimal Prescribing

**Conclusion:**

AOS is an efficient and promising antimicrobial stewardship strategy; provider re-education is needed to increase AOS uptake.

**Disclosures:**

**All Authors**: No reported disclosures.

**Table 2:** Bivariable and Multivariable Regression Analysis of Factors Associated with Optimal Prescribing

†Covaries with intervention group; Hosmer and Lemeshow p = 0.68; Method: backwards logistic regression; No variables removed

